# Effect of Luteolin and Apigenin on the Production of Il-31 and Il-33 in Lipopolysaccharides-Activated Microglia Cells and Their Mechanism of Action

**DOI:** 10.3390/nu12030811

**Published:** 2020-03-19

**Authors:** Denis Nchang Che, Byoung Ok Cho, Ji-su Kim, Jae Young Shin, Hyun Ju Kang, Seon Il Jang

**Affiliations:** 1 Department of Health Management, Jeonju University, Jeonju-si, Jeollabuk-do 55069, Korea; chedenis88@gmail.com (D.N.C.); enzyme21@naver.com (B.O.C.); kim1011003@naver.com (J.-s.K.); sjy8976@naver.com (J.Y.S.); dkgk0608@naver.com (H.J.K.); 2 Department of Food Science and Technology, Chonbuk National University, Jeonju-si, Jeollabuk-do 54896, Korea

**Keywords:** microglia cells, neuro-inflammation, IL-31, IL-33, luteolin, apigenin

## Abstract

Microglia cells are resident cells of the central nervous system (CNS) charged with modulating inflammation in the CNS. Overstimulation of microglia cells continuously releases inflammatory mediators that contribute to neurodegenerative diseases. Apigenin and Luteolin are flavonoids with reported anti-inflammatory activities. However, their effects on IL-31 and IL-33 production in microglial cells are unknown. Here, we investigated the effects of apigenin and luteolin on the production of IL-31 and IL-33 by microglia cells. SIM-A9 microglial cells were pre-treated with apigenin or luteolin and stimulated with lipopolysaccharides to evaluate the production of IL-31 and IL-33. The study revealed that apigenin and luteolin inhibited the production of IL-31 and IL-33 at the gene and protein expressions and the secretion levels. Using potent inhibitors of MAPK, NF-κB, and STAT3 signaling pathways, we demonstrated that apigenin and luteolin’s suppression of ERK and JNK contributed to the inhibition of IL-31 and IL-33 in the MAPK pathway. Luteolin’s suppression of NF-κB and STAT3 also contributed to the inhibition of IL-31 and IL-33. Further analysis revealed that both compounds prevented nuclear translocation of activated NF-κB and STAT3, an act that subsequently prevented their DNA binding activities. Collectively, the study suggested that apigenin and luteolin’s regulation of signaling pathways contributed to the inhibition of IL-31 and IL-33, thus suggesting its importance for the improvement of neurodegenerative diseases involving these two cytokines.

## 1. Introduction

The innate immune defense system of the central nervous system (CNS) has microglia cells as one of the resident cells charged with the duty of modulating inflammation in the CNS to combat external and internal attacks to the CNS. The microglia cells are derived from erythro-myeloid progenitors in the yolk sac during early embryonic development and continuously proliferate to maintain a stable population throughout life [1,2]. Under normal physiologic conditions, the microglia cells promote neural precursor cell proliferation and survival by counteracting any disturbances in immunological homeostasis, which protect neurons that have a limited capacity to regenerate. Under pathological conditions when microglia cells are activated, they play major roles in neurodegenerative diseases including Alzheimer’s disease, Parkinson’s disease, amyotrophic lateral sclerosis, multiple sclerosis, and prion-induced neurodegeneration [3]. They alone or by interacting with other incoming immune cells (acting as antigen-presenting cells) release a vast amount of inflammatory mediators including nitric oxide, prostaglandin E_2,_ IL-1β, IL-6, TNF-α, and as well as reactive oxygen and nitrogen species that caused neuro-toxicity [4].

IL-31 and IL-33 are newly discovered mediators of inflammation that are reported to play a significant role in the pathogenesis of chronic inflammatory diseases including atopic dermatitis, pruritus, inflammatory bowel diseases, and autoimmune diseases such as bronchial asthma, systemic lupus erythematosus, and anaphylactic shock [5,6,7]. In addition, several insights have pointed out on the production of IL-33 in the CNS, suggesting a possible role in neuro-inflammation by recruitment of leukocyte in the brain [8,9]. Consequently, IL-33 deficient mice had decreased lipopolysaccharides (LPS)-induced neuro-inflammation in a study [8]. IL-33 was also found to excite sensory neurons and mediated itch thereby raising the idea that production of IL-33 in the CNS may also mediate itch [10]. The presence of IL-31 receptors in the spinal cord and brain tissues and its association with pruritus also raises the possibilities that cells of the CNS can produce IL-31 to directly stimulate sensory neurons to mediate the itch [11]. Therefore, these reports suggest that the attenuation of all possible cause of neuro-inflammation will be a possible therapeutic or preventive approach for the treatment of diseases involving the CNS.

Flavonoids are a group of multi-functional plant metabolites that are currently being exploited for their therapeutic efficacy for the treatment of a wide range of chronic diseases, including neuro-inflammatory diseases [12,13,14]. They exert pharmacological effects including antioxidants, anti-inflammatory, anti-viral, and can help in the amelioration of metabolic diseases [12,15]. Of these flavonoids, apigenin and its phase I metabolite, naturally present in celery, broccoli, onions, green pepper, parsley, and perilla, have been studied for their biological activities [16]. Apigenin was reported to downregulate cytokines, prostaglandin E, and nitric oxide release and protected Alzheimer’s disease neurons via multiple means by reducing the frequency of spontaneous Ca^2+^ signals and significantly reducing caspase-3/7 mediated apoptosis [17,18]. Luteolin was also found to inhibit microglial inflammation by suppressing the expression of iNOS, COX-2, TNF-α, IL-6, andIL-1β, thereby improving neuron survival against inflammation [19,20,21]. However, there are no reports on the production of IL-31 in microglia and the exact effect of apigenin and luteolin in inhibiting the IL-31 and IL-33 cytokines in the microglia cells. Therefore, in the present study, the effects of apigenin and luteolin in LPS-induced microglial activation and release of IL-31 and IL-33 were investigated in murine microglia SIM-A9 cells. Possible mechanisms of action, as well as DNA binding activities, were determined.

## 2. Materials and Methods

### 2.1. Reagents and Chemicals

Luteolin (≥98% purity) was procured from ChemFaces Wuhan, China. Penicillin/streptomycin antibiotics, Enzyme-linked immunosorbent assay (ELISA) kit IL-31, and antibody against IL-33 were procured from Invitrogen, Carlsbad, CA, USA. Fetal bovine serum and Iscove’s Modified Dulbecco’s Medium (IMDM) were procured from Gibco, Grand Island, NY, USA. Goat anti-mouse IgG (H + L) Alexa Fluor™ plus 488, and Goat anti-rabbit IgG, (H + L) Alexa Fluor™ plus 488 conjugated secondary antibodies came from Invitrogen. Ribospin II extraction kit came from GeneAll Biotechnology, Seoul, Korea. ReverTra Ace^®^ qPCR RT Master Mix with gDNA Remover was procured from Toyobo, Osaka, Japan. SYBR Premix Ex Taq™ was procured from Takara Bio, Nojihigashi, Japan. Bradford’s assay reagent was procured from Bio-Rad Laboratories, Hercules, CA, USA. EZ-western Lumi Pico Alpha and EZ-Cytox reagents were procured from Dogenbio, Seoul, Korea. NE-PER^TM^ nuclear and cytoplasmic extraction reagent and radio-immunoprecipitation assay buffer (RIPA buffer) were procured from Thermo Scientific, Rockford, IL, USA. Polyvinylidene fluoride membrane was procured from Millipore, MA, USA. Antibodies against p-JNK, JNK, p-p38, p38, p-ERK, ERK, p-p65, p65, p-IκB-α, and IκB-α proteins were procured from Santa Cruz Biotechnology, Santa Cruz, CA. Antibodies against IL-31 and ionized calcium-binding adaptor molecule 1 (Iba-1) were procured from Abcam, Cambridge, United Kingdom. Antibodies against actin proteins were procured from Biosciences, Franklin Lakes, NJ, USA. Antibodies against lamin B1, horseradish peroxidase (HRP)-IgG secondary antibodies, and diamidino-2-phenylindole (DAPI) were procured from Cell Signaling Technology, Danvers, MA, USA. Dimethyl sulfoxide and inhibitors of p65, p38, ERK, JNK, and STAT3 and were procured from Sigma-Aldrich, St. Louis, MO, USA. DNA-binding ELISA kits for NF-κB and STAT3 activation were procured from Active Motif, Carlsbad, CA, USA.

### 2.2. Cell Culture

SIM-A9 microglial cells (ATCC, CRL-3265) were grown and maintained in IMDM supplemented with 10% FBS, 5% HS, and 1% penicillin/streptomycin antibiotics in 37 °C under 5% CO_2_ in an incubator. They were maintained in 90 × 20 mm or 160 × 25 mm cell culture dishes until the cells had reached 80% confluence before they were subjected subsequent experiments. The cells were not serum starved in all the experiments performed.

### 2.3. Cell Viability

Microglia cells (3 × 10^5^ cells/mL) were seeded and cultured in 96-well plates for 16 h. The cells were then treated with 0 μM -100 μM of apigenin or luteolin and incubated for a further 24 h, after which EZ-Cytox reagent (10 μM) was added to each well. After 2 h of further incubation, the absorbance of each well was measured using a spectrophotometer (Tecan, Männedorf, Switzerland) at 450 nm. The absorbance of each well corresponded with the microglia cell viability.

### 2.4. RNA Isolation and Quantitative RT-PCR

Microglia cell (5 × 10^5^ cells/mL) were seeded and cultured in 90 × 20 mm for 16 h. The cells were then treated with or without 30 μM or 60 μM of apigenin or luteolin or potent inhibitors of ERK, p38, JNK, p65, or STAT3 and incubated for 1 h, after which LPS, to a concentration of 2 µg/mL was added to stimulate the cells for 3 h. Subsequently, the Ribospin II extraction kit was used to isolate the Total RNA using the manufacturer’s protocol with no modification. The RNA concentration was determined with a spectrophotometer and 2000 ng of total RNA from each sample was reverse transcribed to cDNA using Bio-Rad Thermal Cycler and the ReverTra Ace^®^ qPCR RT Master Mix per the manufacturer’s protocol. Following cDNA synthesis, the cDNA samples were subjected to PCR experiment using Thermal Cycler Dice Real-Time System III and lite (Takara) and SYBR Premix Ex Taq™. The primers used were: GAPDH forward: 5′GGC TAC ACT GAG GAC CAG GT3′ reverse: 5′TCC ACC ACC CTG TTG CTG TA3′ (Accession number: NM_001289726); IL-31 forward: 5′CCT ACC CTG GTG CGT CTT TG3′ reverse: 5′CTG ACA TCC CAG ATG CCT GC3′ (Accession number: NM_029594) and IL-33 forward: 5′TTG GCT TAC GAT GTT G GA3′ reverse: 5′ACT GTG GTG CCT GCT CTT CT3′ (Accession number: NM_001164724). The thermal profile consisted of an initial denaturation at 95 ℃ for 5 min, then 40 cycles at 95 ℃ for 30 s and last by annealing at 62 ℃ for 30 s. All samples were run in triplicate in three separate experiments and the expression levels were normalized with GAPDH using a 2^− ΔΔCt^ comparative method.

### 2.5. Protein Extraction and Western Blot

Microglia cell (5 × 10^5^ cells/mL) were seeded and cultured in 90 × 20 mm for 16 h. The cells were then treated with or without 30 μM or 60 μM of apigenin or luteolin and incubated for 1 h, after which LPS, to a concentration of 2 µg/mL was added to stimulate the cells for 30 min or 12 h. Subsequently, whole proteins were extracted from each treatment samples using protease and phosphatase inhibitors treated-RIPA buffer per the manufacturer’s protocol with no modification. Following protein quantification using Bradford’s assay reagents, 30 µg of proteins from each sample were processed and separated on a 12% or 20% SDS-PAGE with the help of an electrophoresis power supply (100 V for 1 h). Following separation, the proteins were transferred onto polyvinylidene fluoride membranes with the help of the power supply (100 V for 1 h). Next, the membranes were blocked by incubating in 5% bovine serum albumin (BSA) for 1 h and then incubated overnight with various antibodies (Iba1, IL-13, IL-33, p-JNK, JNK, p-p38, p38, p-ERK, ERK, p-p65, p65, p-IκB-α, IκB-α, p-STAT3, STAT3, and actin). After the overnight incubation, the membranes were washed in three changes of TBST solution (5 min each) and further incubated with corresponding HRP-conjugated secondary antibodies for 2 h at room temperature. Next, the membranes were washed in five changes of TBST solution (5 min each) and visualized on a detection Imaging System (Alliance version 15.11; UVITEC Cambridge, UK) with the help of an EZ-western Lumi Pico Alpha chemiluminescence reagent. Stripping buffer was also used in this experiment thereby allowing multiple proteins to be viewed on a single membrane. The band densities were determined using ImageJ analysis software program.

### 2.6. Measurement of Cytokine Production

Microglia cells (5 × 10^5^ cells/mL) were seeded and cultured in 6-well cell culture plates for 16 h. The cells were then treated with or without 30 μM or 60 μM of apigenin or luteolin and incubated for 1 h. Then, LPS to a concentration of 2 µg/mL was added to stimulate the cells for 24 h. The cell culture media was collected, and the concentrations of IL-31 and IL-33 were measured using ELSA kits per the enclosed protocol with no modification.

### 2.7. Nuclear Protein Extraction and Western Blot

Microglia cell (5 × 10^5^ cells/mL) were seeded and cultured in 160 × 25 mm for 16 h. The cells were then treated with or without 30 μM or 60 μM of apigenin or luteolin and incubated for 1 h. Then, LPS to a concentration of 2 µg/mL was added to stimulate the cells for 30 min. Subsequently, nuclear proteins were extracted from each treatment samples using protease and phosphatase inhibitors treated- NE-PER^TM^ nuclear and cytoplasmic extraction reagent per the manufacturer’s protocol with no modification. Following protein quantification using Bradford’s assay reagents, 20 µg of nuclear proteins were used for the western blot experiment, performed as described above. However, in this case, the membranes were incubated with STAT3, p65, or Lamin B. The purity of the nuclear extract was verified by the absence of tubulin in the nuclear extract.

### 2.8. Immunofluorescence

Microglia cell (5 × 10^5^ cells/mL) were seeded and cultured in 4-well cell culture slides for 16 h. The cells were then treated with or without 60 μM of apigenin or luteolin and incubated for 1 h, after which LPS, to a concentration of 2 µg/mL was added to stimulate the cells for 30 min or 12 h. The cells were fixed and permeabilized in ice-cold methanol for 10 min at −20 ℃. After washing in two changes of phosphate-buffered saline (PBS) (5 min each), the cells were incubated in 1% BSA for 1 h to block subsequent non-specific binding. For immunofluorescent staining, the cells were incubated with IL-31, IL-33, p-p65, or p-STAT3 antibodies (in 1% BSA) overnight at 4 ℃. After washing in two changes of PBS (5 min each), the cells were incubated with Alexa Fluor 488-conjugated goat anti-mouse or rabbit IgG secondary antibodies for 1 h at room temperature. Then, the cells were washed and mounted with DAP mounting medium and photographed under a ZEISS Confocal Microscopy (Oberkochen, Germany).

### 2.9. NF-κB and STAT DNA Binding Activity Assay

Microglia cells (5 × 10^5^ cells/mL) were seeded and cultured in 160 × 25 mm for 16 h. The cells were then treated with or without 60 μM of apigenin or luteolin and incubated for 1 h. Then, LPS to a concentration of 2 µg/mL was added to stimulate the cells for 30 min. Nuclear proteins were extracted and measured as described above. NF-κB and STAT3 activation kit was used to perform the NF-κB-DNA and STAT3-DNA binding activity according to the manufacturer’s guidelines with little modification. Briefly, 10 μg of nuclear extract was added to 0.03 mL of complete binding buffer in the well plates pre-coated with NF-κB and STAT3 DNA targets. The plate was incubated with mild agitation for 1 h at room temperature. Following washing of the plate, NF-κB or STAT3 antibodies (0.1 mL) that recognizes only epitopes of p65 and STAT3 (respectively) that are bound to DNA in the wells was added to each well and further incubated for at room temperature for 1 h. The wells were then washed and 0.1 mL of diluted HRP, and conjugated antibodies were added to each well. The plates were incubated for 1 h at room temperature, washed, and 0.1 mL of the developing solution was added into the wells for 10 min. The reaction was stopped by adding 0.1 mL of stop solution. The absorbance was read at 450 nm and this absorbance corresponded with the DNA binding activities.

### 2.10. Statistical Analysis

The data are presented as the mean ± standard deviation (*n* = 3). The data obtained for the treated or non-treated cells were a little skewed and kurtotic, and the Shapiro-Wilk tests all had a p-value of greater than 0.05, thus indicating that our data were approximately normally distributed. Hence, one-way analysis of variance (ANOVA) followed by Duncan’s multiple comparison test was used to compare the different treated or non-treated cells. Statistical significance was defined when the p-value was less than 0.05.

## 3. Results

### 3.1. Effects of Apigenin, Luteolin, and LPS on Cytotoxicity to Microglia Cells

**Figure 1:**
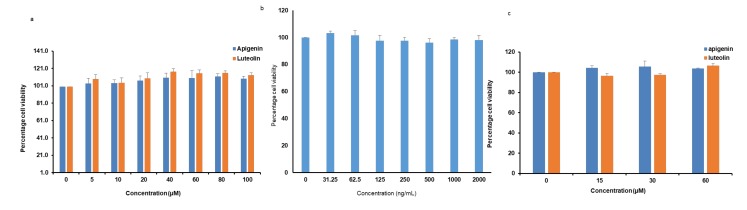
Apigenin, luteolin, and lipopolysaccharides (LPS) showed no cytotoxicity to the microglial cell. Microglial cells (5 × 10^5^ cells/mL) were cultured and treated with apigenin or luteolin at indicated concentrations (**a**); LPS at indicated concentrations (**b**); and apigenin or luteolin at 0, 15, 30, and 60 µM and treated with 2000 ng/mL (**c**) for 24 h before cell viability studies were performed. The results are presented as the mean ± standard deviation (SD) (*n* = 3).

Water Soluble Tetrazolium Salts (WST) assay was used to evaluate the cytotoxic effects of apigenin and luteolin on microglia cells. When the cells were treated with apigenin or luteolin at different concentrations of 0, 5, 10, 20, 40, 60, 80, and 100 µM, no significant cytotoxicity to the microglia cells up to 100 µM was observed (Figure 1a). To rule out further cytotoxicity issues, we also confirmed that LPS up to 2 µg/mL did not have cytotoxic effects on the microglia (Figure 1b) and that co-treatment with apigenin or luteolin (up to 60 µM) and LPS (2 µg/mL) did not also have cytotoxicity issues on the microglia cells (Figure 1c). Based on these findings, the concentration of 30 and 60 µM of apigenin or luteolin and LPS (2 µg/mL) was chosen for subsequent experiments.

### 3.2. Effects of Apigenin and Luteolin on Iba-1 Expression in Stimulated Microglia

**Figure 2:**
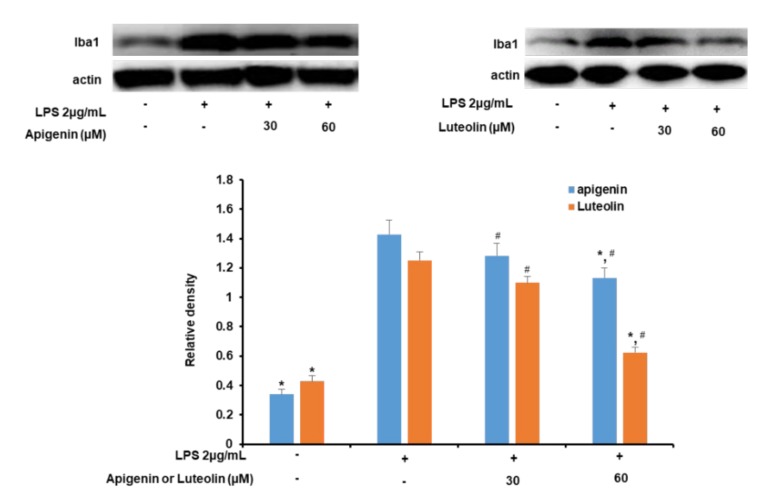
Apigenin and luteolin inhibit LPS-induced expression of Iba1 in microglial cells. Microglia cells (5 × 10^5^ cells/mL) were cultured and pre-treated with apigenin or luteolin at the concentrations of 30 and 60 µM and then stimulated with LPS for 30 min. The expression level of Iba1was investigated by western blot assay. The band densities were analyzed using ImageJ analysis software, with respect to actin. The results are presented as the mean ± SD (*n* = 3). The asterisk (*) indicates significant different at *p* < 0.05 compared to the LPS-only treated cells for bars with the same color. The hash (#) indicates a significant difference at # *p* < 0.05 for bars with different colors within the same treatment concentration.

Iba-1 expression was measured to investigate the effects of the apigenin and luteolin treatment on LPS-induced microglia activation. As shown in Figure 2, treatment of microglia cells with LPS significantly increased Iba1 expression in the cells 30 min after microglia stimulation. However, when the cells were pre-treated with apigenin or luteolin before stimulation with LPS, the expression of the Iba-1 was decreased as the concentration of apigenin or luteolin treatments increased from 30 to 60 µM. Apigenin and luteolin significantly decreased Iba-1 expression at 60 µM.

### 3.3. Effects of Apigenin and Luteolin on IL-31 and IL-33 mRNA and Protein Expression in Stimulated Microglia

**Figure 3:**
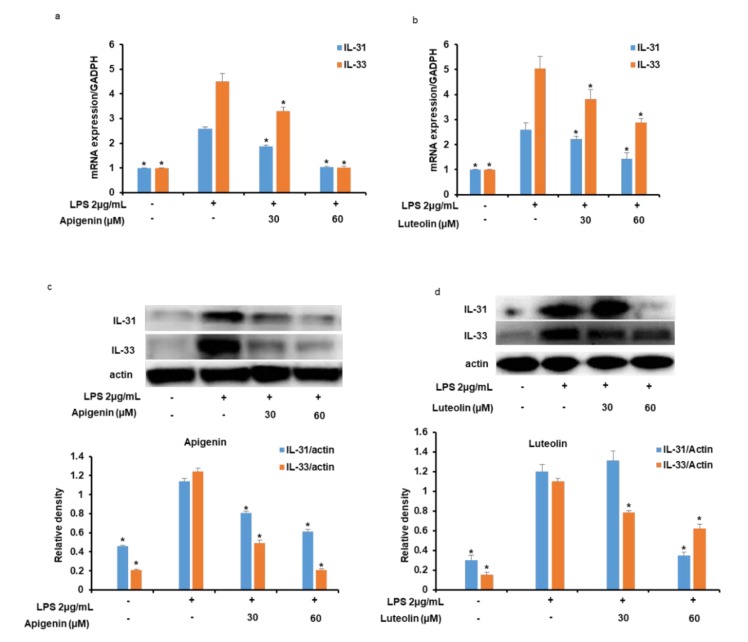
Apigenin and luteolin inhibit LPS-induced intracellular expression of IL-31 and IL-33 mRNA and protein expressions. Microglia cells (5 × 10^5^ cells/mL) were cultured and pre-treated with apigenin or luteolin at the concentrations of 30 and 60 µM and then stimulated with LPS for 3 h (**a**,**b**) or 12 h (**c,d**). The mRNA expressions of IL-31 and IL-33 were investigated by quantitative real-time PCR analysis (**a,b**). The protein expression levels of IL-31 and IL-33 was investigated via western blot assay and the band densities were analyzed using ImageJ analysis software, with respect to actin (**c,d**). The results are presented as the mean ± SD (*n* = 3). The asterisk (*) indicates a significant difference at *p* < 0.05 compared to the LPS-only treated cells for bars with the same color. GADPH: Glyceraldehyde 3-phosphate dehydrogenase.

**Figure 4:**
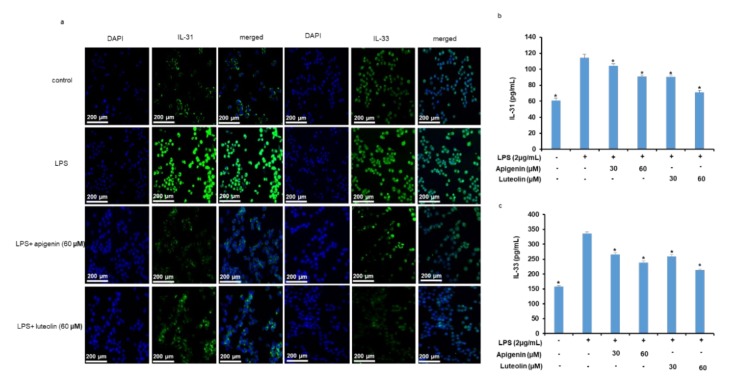
Apigenin and luteolin inhibit LPS-induced expression of IL-31 and IL-33 protein expressions and their secretion out of microglia cells. Microglia cells (5 × 10^5^ cells/mL) were cultured and pre-treated with apigenin or luteolin and then stimulated with LPS for 12 h (**a**) or 24 h (**b,c**). The expression of IL-31 and IL-33 in the microglia was investigated by immunofluorescence at ×10 magnification (**a**). The Cell media were collected for IL-31 and IL-33 measurements by ELISA assay kits (**b,c**). Where applicable, the results are presented as mean ± SD (*n* = 3). The asterisk (*) indicates a significant difference at *p* < 0.05 compared to the LPS-only treated cells for bars with the same color.

As shown in Figure 3a,b, treatment of microglia cells with LPS significantly increased the mRNA expressions of IL-31 and IL-33 in the cells 3 h after the microglia stimulation. However, when the cells were pre-treated with apigenin (Figure 3a) or luteolin (Figure 3b) before stimulation with the LPS, the expression of the IL-31 and IL-33 mRNAs was significantly decreased as the concentration of apigenin or luteolin was increased from 30 to 60 µM. Additionally, stimulation of the microglia cells with LPS significantly increased the intracellular protein expressions of IL-31 and IL-33 in the cells 12 h after the microglia stimulation (Figure 3c,d). However, when the cells were pre-treated with apigenin (Figure 3c) or luteolin (Figure 3d) before stimulation with the LPS, the intracellular expression of the IL-31 and IL-33 proteins was significantly decreased with apigenin or luteolin treatments at the different treatment doses. In addition, the effects of apigenin or luteolin treatment on intracellular IL-31 and IL-33 protein expression was also verified by immunofluorescence staining. The results did confirm that LPS significantly stimulated the expressions of IL-31 and IL-33 in the activated microglia cells and that apigenin and luteolin effectively suppressed the expressions of the IL-31 and IL-33 protein expressions in microglia cells (Figure 4a).

### 3.4. Effects of Apigenin and Luteolin on IL-31 and IL-33 Secretion In Stimulated Microglia

As shown in Figure 4b,c, stimulation of the microglia cells with LPS resulted to a significant increase in the concentrations of IL-31 (Figure 4b) and IL-33 (Figure 4c) in the culture media of the microglia cells indicating the secretion of both cytokines out of the microglia cells. However, when the cells were pre-treated with apigenin or luteolin before stimulation with the LPS, the concentrations of IL-31 and IL-33 was found to be significantly decreased in a dose-dependent manner.

### 3.5. Effects of Apigenin and Luteolin on MAPK Pathway in Stimulated Microglia

**Figure 5:**
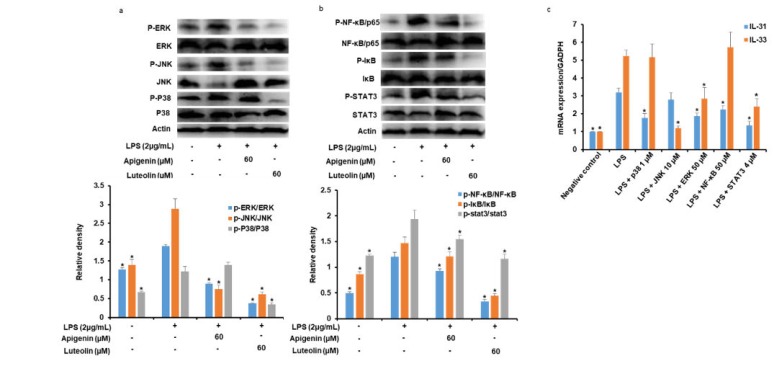
Apigenin and luteolin regulate the activation of mitogen-activated protein kinase (MAPK), NF-κB, and STAT signal pathways in LPS-induced microglial activation. Microglia cells (5 × 10^5^ cells/mL) were cultured and pre-treated with apigenin or luteolin (**a,b**) or p38, JNK, ERK, NF-κB, or STAT3 potent inhibitors (**c**) and then stimulated with LPS for 30 min. The protein expression levels of cell signaling kinases were investigated by western blot assay and the band densities were analyzed using ImageJ analysis software, with respect to actin (**a,b**). The mRNA expression levels of IL-31 and IL-33 were investigated by quantitative real-time PCR analysis (**c**). The results are presented as the mean ± SD (*n* = 3). The asterisk (*) indicates a significant difference at * *p* < 0.05 compared to the LPS-only treated cells for bars with the same color.

As shown in Figure 5a, LPS treatment for 30 min facilitated the activation of ERK, JNK, and P38 to p-ERK p-JNK, and p-P38, respectively, and specifically caused the degradation of JNK. However, when the cells were pre-treated with apigenin or luteolin before stimulation with the LPS, the microglia cells pre-treated with apigenin showed a significant reduction in p-ERK and p-JNK expression and no significant effects of p-P38 expression. The microglia cells pre-treated with luteolin showed a significant reduction of p-ERK, p-JNK, and p-P38 expressions.

### 3.6. Effects of Apigenin and Luteolin on NF-κB and STAT3Ppathways in Stimulated Microglia

As shown in Figure 5b, LPS treatment for 30 min facilitated the phosphorylation of NF-κB/p65, IκB, and STAT3 (p-NF-κB/p65, p-IκB, and p-STAT3, respectively). However, when the cells were pre-treated with apigenin or luteolin before stimulation with the LPS, the microglia cells pre-treated with apigenin showed a significant reduction in p-NF-κB/p65, p-IκB, and p-STAT3 expressions. Similarly, the microglia cells pre-treated with luteolin also showed a significant reduction of p-NF-κB/p65, p-IκB, and p-STAT3 expressions.

### 3.7. Effects of Potent Inhibitors of MAPK, NF-κB, and STAT3 Pathways on IL-31 and IL-33 Production in Stimulated Microglia

As reported in Figure 5c, stimulation of microglial cells with LPS significantly increased the mRNA expression of IL-31 and IL-33 in the microglia cells 3 h after stimulation. However, when the cells were pre-treated with ERK, JNK, P38, NF-κB/p65, and STAT3 potent inhibitors, before stimulation with the LPS, the mRNA expressions of the IL-33 was significantly decreased in the JNK, ERK, and STAT3 inhibitor-treated cells, while IL-31 mRNA was significantly decreased in ERK, P38, NF-κB/p65, and STAT3 inhibitor-treated cells.

### 3.8. Effects of Apigenin and Luteolin on NF-κB/p65 and STAT3 Nuclear Translocation and DNA Binding Activities in Stimulated Microglia

**Figure 6:**
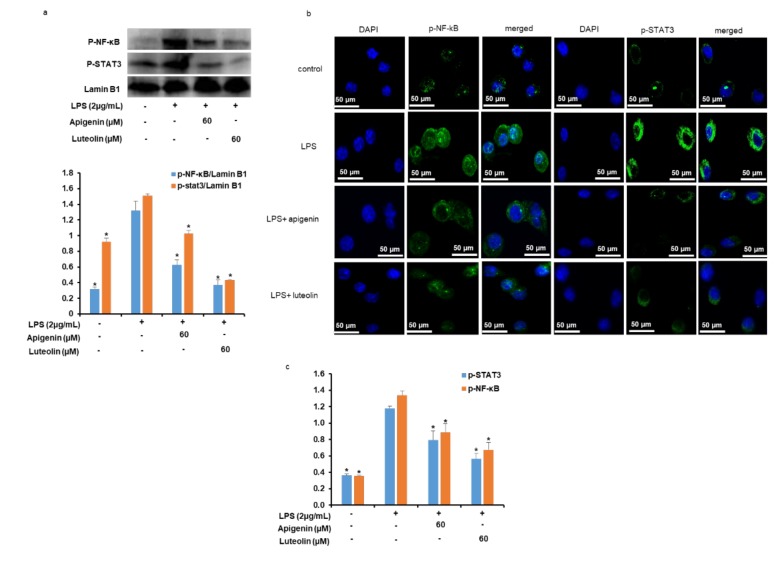
Apigenin and luteolin inhibited the nuclear translocation of NF-κB and STAT and their DNA binding activities. Microglia cells (5 × 10^5^ cells/mL) were cultured and pre-treated with apigenin or luteolin before the cells were stimulation with LPS for 30 min. The protein expression levels of activated NF-κB and STAT in nuclear extract were investigated by western blot assay and the band densities were analyzed using ImageJ analysis software, with respect to lamin B1 (**a**). The expression of activated NF-κB and STAT translocation to the nucleus of microglial were investigated by immunofluorescence at ×40 magnification (**b**). The DNA binding activities of activated NF-κB and STAT were evaluated in the nuclear extracts using the ELISA-based method (**c**). Where applicable, the results are presented as the mean ± SD (*n* = 3). The asterisk (*) indicates a significant difference at * *p* < 0.05 compared to the LPS-only treated cells for bars with the same color.

As reported in Figure 6a and b, the stimulation of microglial cells with LPS for 30 min significantly induced the translocation of phosphorylated NF-κB/p65 and STAT3 into the nucleus as depicted by the increase in the nuclear expression of NF-κB/p65 and STAT3 (Figure 6a). However, when the cells were pre-treated with apigenin or luteolin before stimulation with LPS, the expression of p-NF-κB/p65 and p-STAT3 in the nuclear extracts were significantly decreased. To confirm the nuclear translocation of p-NF-κB/p65 and p-STAT3, immunofluorescence staining of the apigenin or luteolin-treated stimulated microglia cells was performed. The data obtained confirmed the decrease in p-NF-κB/p65 and p-STAT3 nuclear translocation with apigenin or luteolin treatment (Figure 6b). p-NF-κB/p65 and p-STAT3 DNA binding activity data further revealed that while stimulation of the microglia cells with LPS increased the p-NF-κB/p65 and p-STAT3 DNA binding activities, pretreatment with apigenin or luteolin significantly decreased the DNA binding activities of p-NF-κB/p65 and p-STAT3 (Figure 6c).

## 4. Discussion

LPS is known to induce neurotoxic mediators such as nitric oxide, PGE_2_, and an array of pro-inflammatory cytokines by activating microglial cells and causing neuronal damage [22,23]. In this study, we found that LPS also activated the microglia cells as Iba-1, a well-known microglial activation marker was found to be overexpressed in the LPS-treated cells. The overexpression of this marker was suppressed by apigenin and luteolin thus implying that the two flavonoids suppressed the activation of LPS-induced microglial activation. The result is similar to another study that reported that apigenin and luteolin modulated microglial activation by decreasing the expression of IFN-γ-induced microglial CD40 [24]. The study further reviews that apigenin and luteolin suppressed the production of IL-31 and IL-33 by inhibiting their gene and protein expressions in the microglia cells, and finally inhibiting their secretion out of the microglial cells. The ability of apigenin and luteolin to regulate LPS-induced microglia activation and subsequent pro-inflammatory IL-31 and IL-33 mediators may be useful in the treatment of inflammatory diseases in the CNS including Alzheimer’s disease, malaria infections, itch caused by the release of itch mediator within the CNS, and autoimmune diseases like multiple sclerosis, where there is a positive correlation with IL-31 or IL-33 [6,25,26,27]. The data adds more knowledge to the current understanding of the anti-inflammatory activities of these two flavonoids that have also been previously reported to be neuroprotective by modulating other inflammatory mediators [17,28,29,30]. Although IL-31 receptors have been reported in the spinal cord and brain tissues, no study has reported on the production or expression of IL-31 in the CNS. In the present study, we have reported the expression of IL-31 mRNA and proteins in the CNS transformed cell line (microglia). The fact that IL-31 is specifically associated with pruritus in atopic dermatitis and the expression of IL-31 receptors in the CNS raises the suggestion that the pruritus caused by IL-31 might also arise within the CNS itself. Interestingly, activated astrocyte, another immune cell of the CNS, has been reported as new players of chronic itch in atopic dermatitis by releasing itch mediators [31]. Although IL-33 has detrimental effects on the brain, complete suppression of in the CNS may prove to be hazardous, as IL-33 has an important role in promoting the myelination and repair of demyelinated neurons; regulation of the development and maturation of neuronal circuits and the entire brain development [32,33]. Rather, modulation of IL-33 production in CNS would appear to be the most appropriate course of action for treating neurodegenerative diseases involving IL-33. Our data show that apigenin and luteolin at high concentrations can modulate IL-33 production, thus implying that the concentration of apigenin and luteolin is an important aspect to consider in rendering beneficial effects and minimizing detrimental effects. This modulation is often quite difficult to achieve with conventional drugs.

To understand the molecular mechanism of action of apigenin and luteolin in inhibiting IL-31 and IL-33 expression in LPS-stimulated microglial cells, we studied the effects of the two flavonoids on signaling pathways that are responsible for signaling the production of pro-inflammatory cytokines of most immune cells including microglia cells. MAPKs (ERK, JNK, and p38) activations by phosphorylation is observed in LPS-stimulated microglia cells whereby the can activate transcription factors including NF-κB that will mediate the production of neuroinflammatory mediators such as NO, PGE_2,_ and inflammatory cytokines [34,35,36]. Our study revealed that apigenin suppressed the phosphorylation activation of ERK and JNK and did not affect p38 activation, while luteolin suppressed the activation of all the three MAKPs. Using potent inhibitors of ERK, JNK, and p38, we demonstrated that only ERK and p38 was partly responsible for the production of IL-31 and that only JNK and ERK were partly responsible for the production of IL-33 in the LPS-stimulated microglia cells. The results suggest the implication of the MAPK signaling pathway in the suppressive effects of apigenin and luteolin on IL-31 and IL-33 production in LPS-stimulated microglia cells by activating different kinases of the pathway. Other pathways investigated in this study was the NF-κB and STAT3 signaling pathways. The NF-κB signaling pathway was also shown to stimulate the expression of inflammatory genes such as NO, PGE_2_, and inflammatory cytokines in immune cells of the CNS [37,38]. The phosphorylation of IκB causing its activation and separation from NF-κB activates the NF-κB and causes its translocation to the nucleus and subsequent binding to its promoter region of DNA molecules, thus regulating gene expressions. Signal transduction involving the activation of transcription STAT3 has been shown in activated microglia cells where they mediate the expression of pro-inflammatory responses, as activated STAT3 can translocate to the nucleus and can bind to DNA [39]. In our study, apigenin and luteolin suppressed the activation of NF-κB/p65 probably through its action in causing the degradation of IκB from NF-κB. Apigenin and luteolin also suppressed the activation of STAT3. The fact that potent inhibitors of NF-κB and STAT3 did inhibit the expression of IL-31 mRNA and that only STAT3 inhibitors inhibited IL-33 mRNA suggests that these two pathways in addition to the MAPKs pathways also mediate the production of IL-31 and IL-33 in LPS-activated microglia cells. Further findings revealed that apigenin and luteolin also inhibited the translocation of NF-κB/p65 and STAT3 from the cytoplasm to the nucleus and subsequently affected their DNA-binding activities. The results offer another potential mechanism of action of apigenin and luteolin on IL-31 and IL-33 production in LPS-stimulated microglia cells by inhibiting the activation, translocation, and DNA-binding activities of NF-κB/p65 and STAT3.

It is quite clear from the above results that apigenin and luteolin are effective immunomodulators, although their immediate molecular targets are still unknown. However, these compounds can certainly interact with MAPK, NF-κB, and STAT3 signaling pathways and consequently prevent the transcription of its target genes. It is even more likely that these compounds might act further upstream to exhibits its effect at the DNA-binding levels. In this light, it will be important to investigate other upstream kinases in microglia cells as potential immune-regulatory signaling molecules to further understand the molecular mechanism of action of these two compounds.

In conclusion, IL-31 can also be released from microglia cells in small quantities that can be pathologically important. Apigenin and luteolin by acting on MAPKs, NF-κB, and STAT3 signaling pathways in LPS-activated microglial cells can suppress the expression of IL-31 and IL-33 at the mRNA and protein levels and subsequently inhibits their secretion out of the microglia cells which can affect their downstream signaling. Apigenin and luteolin can further be considered for the treatment of neuro-immune diseases associated with IL-31 and IL-33. These facts will need to be demonstrated in in-vivo studies.
